# Applications of Interpedicular Distance and Anteroposterior Diameter in the Approximation of the Spinal Canal Area

**DOI:** 10.7759/cureus.48747

**Published:** 2023-11-13

**Authors:** Zachary Brandt, Jacob Razzouk, Kai Nguyen, Mark Oliinik, Patricia Carlson, Andrew J Cabrera, Alex Bouterse, Emily Novak, Asael Isaac, Juliette Scolieri, Mei Carter, Olumide Danisa, Wayne Cheng

**Affiliations:** 1 School of Medicine, Loma Linda University, Loma Linda, USA; 2 Orthopedic Surgery, Loma Linda University Medical Center, Loma Linda, USA; 3 Orthopedic Surgery, Jerry L. Pettis Veterans Affairs (VA) Medical Center, Loma Linda, USA

**Keywords:** canal area, diameter, lumbar spine, spinal canal, interpedicular distance, artificial intelligence, machine learning

## Abstract

Introduction: Advancements within the field of medicine revolve around increasing the efficiency of diagnosing and subsequently treating patients. One such advancement is measurements of the central canal using artificial intelligence (AI). The authors propose the possibility of AI measuring two linear distances followed by a subsequent approximation via an area equation. The lumbar spinal canal was approximated by an area calculation using the interpedicular distance (IPD) and anteroposterior diameter (AP diameter). The three shapes evaluated were an ellipse, triangle, and rectangle.

Methods: IPD, AP diameter, and spinal canal area from L1-L5 were measured in 555 patients using the IMPAX6 (Mortsel, Belgium: Agfa-Gevaert) picture archiving and communication system. Subsequently, an approximated area of the lumbar spinal canal, assuming an ellipse shape, was calculated using ellipse equation/approximation. Triangular and rectangular approximations were done using triangle equation/approximation and rectangle equation/approximation, respectively. The equations used are the geometric equations for the area of each shape described. For example, the triangular approximation used the IPD as the base of the triangle and the AP diameter as the height. Thus, the area approximation was calculated by half of the IPD times the AP diameter.

Results: The percent error of the ellipse approximation was the lowest with a range of error from 8.44% at L1 to 15.51% at L5. The triangle approximation again was the second most accurate with a range of error starting at -26.46% at L5 to -30.96% at L1. Lastly, the percentage errors of the rectangle approximation began at 38.07% at L1 to 47.07% at L5. The ellipse and rectangle approximation consistently overestimated the area of the spinal canal, while the opposite was true for the triangle approximation. A combination of these approximations could be used to construct a second-order approximation. The approximations were all highly correlated with the authors’ manual measurements. Approximations at the L2 vertebrae were highest with a correlation of 0.934 closely followed by all approximations at L5 with a value of 0.931. Approximations were least correlated with the L4 vertebrae with a value of 0.905.

Conclusion: The correlation between the approximation equations and the measured values is significantly related. The ellipse equation best predicted the area of the spinal canal followed by the triangle and then the rectangle approximation. The percent error difference of the ellipse approximation at L1 was similar in error compared to other causes of measurement error. Continued investigation into a second-order approximation may yield a more accurate approximation.

## Introduction

Lumbar spinal stenosis is common, affecting approximately 11% of older adults in the United States [1]. Lumbar spinal stenosis does not always require operative treatment, however, decompression is usually associated with good or excellent outcomes in 80% of patients [2]. Canal stenosis is now the most common indication for lumbar spine surgery in elderly subjects [3]. Lumbar spinal stenosis is diagnosed via the clinical picture alongside imaging modalities, such as X-ray, magnetic resonance imaging (MRI), and computed tomography (CT) [1]. Although the accuracy of these imaging modalities varies on sources, the process starts with approximating the area of the spinal canal [4,5].

Advancements within the field of medicine revolve around increasing the efficiency of diagnosing and subsequently treating patients. One such advancement is measurements of the central canal using artificial intelligence (AI). There are a variety of methods in which this can be accomplished. One of which includes AI directly measuring the area of the lumbar spinal canal [6]. The authors propose an alternative possibility of AI measuring two linear distances followed by a subsequent approximation via an area equation. The reason for this proposal is that while it is possible to determine the area of an irregular entity, like the lumbar spinal canal, there are certain limitations involved. The process of training AI to measure the central canal can be quite burdensome, especially regarding the accuracy of measurements [6]. Challenges in achieving accurate measurements revolve around the need to repeat multifactorial scenarios. This entails reducing noise in the initial images and ensuring consistent presentation to minimize the margin of error [7,8]. Maintaining this repeatability of imaging quickly becomes difficult with a variety of imaging modalities and methods. An alternative method, as proposed in this paper, would be to use AI-generated linear measurements with subsequent equation approximation to calculate the area of the lumbar spinal canal. AI-generated linear measurements have displayed a high degree of accuracy as cephalic measurements demonstrated a 98.95% exact replication of gold standard measurements with 0 mm of error [9]. The first step within this process is proving the validity of an approximation equation. It is necessary to show the accuracy of an equation approximation before it can be used within the context of AI. It is proposed that the shape of the spinal canal can be approximated by an area calculation using the two linear measurements of interpedicular distance (IPD) and anteroposterior (AP) diameter. The three shapes evaluated were an ellipse, triangle, and rectangle.

## Materials and methods

Following Loma Linda University Institutional Review Board (IRB) approval (#5230005), we reviewed medical and radiographic records of patients between 18 and 35 years of age who received abdomen and pelvis or lumbar CT between January 2015 and March 2023. Patient consent was not required due to the nature of this retrospective, radiographic study. All patients for possible inclusion in the study were reviewed in a systematic order corresponding to the chronological sequence in which their imaging was completed. Exclusion criteria were composed of patients with a history of disc degeneration, scoliosis with a measured coronal deformity greater than 10 degrees, spondylolisthesis, traumatic spinal injury, infection, malignancy, back or leg pain or numbness, existing spinal hardware, or previous spinal surgery.

Measurement technique

**Figure 1:**
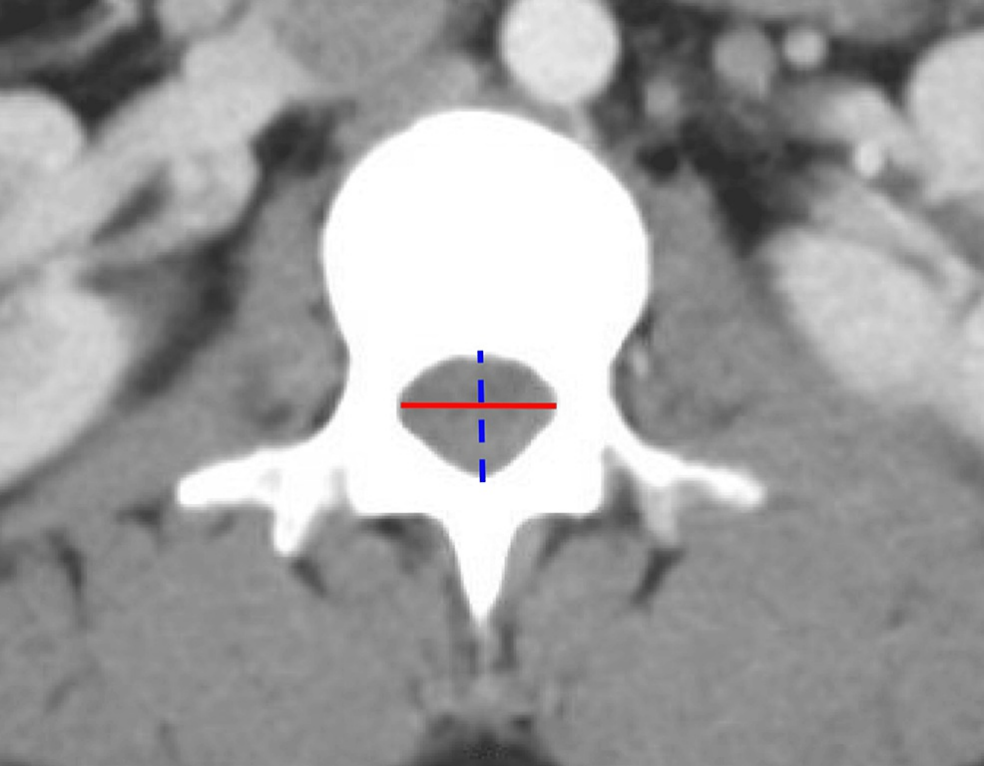
Measurements of IPD (red, solid line) and AP diameter (blue, dashed line).

**Figure 2:**
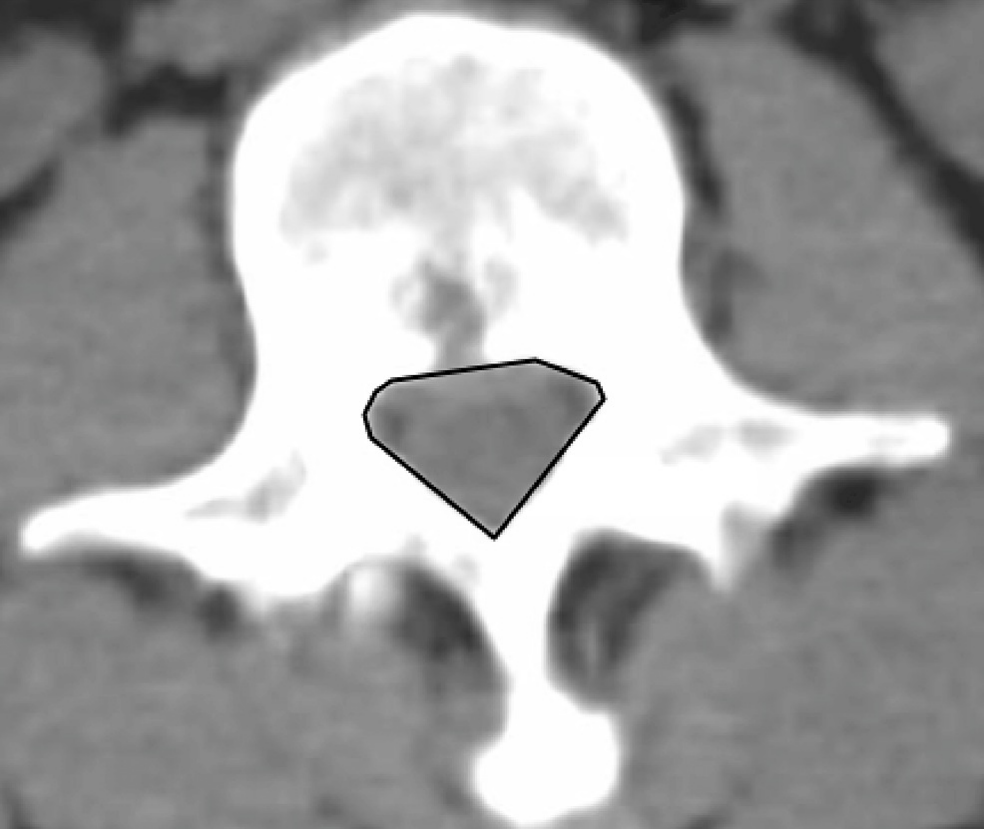
Measurement of spinal canal area.

IPD, AP diameter, and spinal canal area from L1-L5 were measured in 555 patients using the IMPAX6 (Mortsel, Belgium: Agfa-Gevaert) picture archiving and communication system. Window and level designations of 2,000 Hounsfield unit (HU) and 500 HU were used, respectively. All measurements were performed by medical students trained by a board-certified neuroradiologist (NW). Measurements of IPD and AP diameter are demonstrated in Figure 1, and the area of the spinal canal is demonstrated in Figure 2.

IPD was defined as the maximum distance between the medial aspect of the pedicles at a given vertebral level. Canal area was measured using the IMPAX6 markup freeform tool. Subsequently, an approximated area of the lumbar spinal canal, assuming an ellipse shape, was calculated using Equation 1. Triangular and rectangular approximations were done using Equations 2 and 3, respectively. The equations used are the geometric equations for the area of each shape described. For example, the triangular approximation used the IPD as the base of the triangle and the AP diameter as the height. Thus, the area approximation was calculated by half of the IPD times the AP diameter.

Equation 1: Ellipse Approximation

Area = $\pi \times \frac{1}{2}\times (IPD) \times \frac{1}{2}\times (diameter)$

Equation 2: Triangle Approximation

Area = $\frac{1}{2}\times (IPD) \times (diameter)$

Equation 3: Rectangle Approximation

 Area = $(IPD) \times (diameter)$

Statistical analyses

SPSS version 28 (Armonk, NY: IBM Corporation) was utilized for all statistical analyses with alpha defined as p<0.05. Kolmogorov-Smirnov tests and Q-Q plots were used to assess data normality and Levene’s homogeneity of variance test and regression residual plots were used to evaluate homoscedasticity. Pearson correlation tests were used to assess associations between anatomic measurements and patient anthropometric factors, with coefficients categorized as weak, moderate, and strong corresponding to value ranges of 0-0.4, 0.4-0.7, and 0.7-1, respectively. Descriptive statistics consisted of means, standard deviations (SD), ranges, mean differences (MD), and 95% confidence intervals (CI). A paired sample t-test was performed to assess the significance of the difference between measured and approximated values. In addition, mean differences along with percent error calculations were computed. Finally, a Pearson correlation coefficient between approximated and measured areas was calculated.

## Results

A total of 555 patients were evaluated in this study, of which 231 were male and 324 were female. Mean age was 27.2 years, with a range of 18-35 years. Mean height was 1.54±0.09 m, with a range of 1.17 to 1.92 m. Mean weight was 80.44±23.42 kg, with a range of 35.42-209.22 kg. Mean BMI was 28.27±7.2 kg/m², with a range of 15.33-62.89 kg/m².

The average area of the spinal canal by the authors’ manual measurements ranged from 253.28 mm² at L3 to 318.50 mm² at L5. The average area of the spinal canal at each lumbar vertebrae is listed in Table 1. The ellipse approximation most closely approximated the area of the spinal canal with a range of mean differences from 23.10 mm² in L2 to 49.41 mm² in L5. The triangle calculation was the second most accurate approximation with a range of mean differences from -73.75 mm² in L4 to -87.25 mm² in L1. Lastly, the rectangular approximation has the greatest mean differences ranging from 149.93 mm² in L5 to 100.48 mm² in L3. All measured differences were statistically significant (p<0.001). All mean differences are shown in Table 2.

**Table 1:** Mean measurements for approximation of canal area using manual measurement, ellipse, triangle, and rectangle approximations. The data has been represented as mean±SD.

Level	Manual measurement	Ellipse	Triangle	Rectangle
L1	281.78±48.89	305.56±56.50	194.53±35.97	389.06±71.93
L2	262.25±49.79	285.35±57.05	181.66±36.32	363.32±72.64
L3	253.28±49.93	277.85±58.15	176.88±37.02	353.76±74.04
L4	266.16±64.16	302.24±75.08	192.41±47.80	384.82±95.60
L5	318.50±90.23	367.91±100.59	234.22±64.03	468.43±128.07

**Table 2:** Mean differences compared to manual measurement of spinal canal area. Mean differences are represented as mean±SD.

Level	Shape	Mean difference (mm²)	p-Value	Correlation (r)
L1	Ellipse	23.78±23.93	<0.001	0.907
	Triangle	-87.25±22.24	<0.001	0.907
	Rectangle	107.28±34.45	<0.001	0.907
L2	Ellipse	23.10±20.64	<0.001	0.934
	Triangle	-80.59±20.47	<0.001	0.934
	Rectangle	101.07±31.58	<0.001	0.934
L3	Ellipse	24.57±24.47	<0.001	0.909
	Triangle	-76.40±22.47	<0.001	0.909
	Rectangle	100.48±35.47	<0.001	0.909
L4	Ellipse	36.08±32.15	<0.001	0.905
	Triangle	-73.75±29.15	<0.001	0.905
	Rectangle	118.66±46.40	<0.001	0.905
L5	Ellipse	49.41±36.88	<0.001	0.931
	Triangle	-84.28±38.53	<0.001	0.931
	Rectangle	149.93±55.02	<0.001	0.931

The percent error of the ellipse approximation was the lowest with a range of error from 8.44% at L1 to 15.51% at L5. The triangle approximation again was the second most accurate with a range of error starting at -26.46% at L5 to -30.96% at L1. Lastly, the percentage errors of the rectangle approximation began at 38.07% at L1 to 47.07% at L5. All percent error values are in Table 3.

**Table 3:** Approximation percent error calculations compared to manual measurements.

Level	Ellipse	Triangle	Rectangle
L1	8.44%	-30.96%	38.07%
L2	8.81%	-30.73%	38.54%
L3	9.70%	-30.16%	39.67%
L4	13.56%	-27.71%	44.58%
L5	15.51%	-26.46%	47.07%

The approximations were all highly correlated with the authors’ manual measurements. Approximations at the L2 vertebrae were highest with a correlation of 0.934 closely followed by all approximations at L5 with a value of 0.931. Approximations were least correlated with the L4 vertebrae with a value of 0.905. All correlation values are in Table 2.

## Discussion

The choice to use a two-linear measurement approximation stems from the accuracy observed in studies on AI anatomic approximations [9]. From these two measurements, a variety of shapes could be calculated. The shapes used for approximation were chosen for two reasons. Firstly, due to literature that previously described the shape of the spinal canal [10]. Secondly, shapes that could consistently overestimate area or underestimate area in the hope of investigating a second-order approximation. The effectiveness of the authors’ approximation of the spinal canal is multifactorial. The factors to consider to evaluate the approximation are how well the approximation follows described patterns, how consistent the error of the approximation is, and critically, how accurate the approximation is to gold standard values.

The approximation used revolves around two measurements. These two measurements have previously described patterns that affect both the raw measurements and approximations. Within the caudal lumbar spine, the AP diameter should increase and a failure to do so can be used to assist in stenosis diagnosis [11,12]. Similarly, the interpedicular distance has been described to increase from L1 to L5 [13]. The area of the spinal canal in the lumbar spine exhibits an interesting pattern of decreasing in area to a minimum at L3 and then increasing in area to L5 [14]. This phenomenon is replicated by measured data and each of our approximations. The ellipse, triangle, and rectangle each decrease from L1 to L3 and then subsequently increase from L3 to L5. These values can be observed in Table 1.

As could be predicted based on previous literature, the approximation of the ellipse most accurately replicated the area of the spinal canal [10]. This approximation consistently overestimated the area of the spinal canal which was similar to the rectangle approximation. The rectangle approximation overestimated the area of the spinal canal by large margins, however, it may still have utility in a second-order approximation. The triangle approximation consistently underestimated the area of the spinal canal making a second-order approximation possible with currently calculated shape approximations. A second-order approximation would use a ratio of the authors’ currently calculated approximation to find a value of greater fit. These approximations are common in fields of engineering and other math-based fields [15]. This would be done by taking a ratio of the ellipse or rectangle approximation, approximations that overestimate the area, and combining that with a ratio of the triangle approximation which underestimates the area. Additional mathematical analysis would need to be conducted to test the validity of this purposed second-order approximation, however, for each approximation to consistently either overestimate or underestimate area makes this possible.

The most critical assessment of each of the shape approximations is to evaluate the accuracy compared to the manually measured values. Each approximation at each vertebrae level was statistically significant from the manually measured values (p<0.001). This was to be expected due to the sheer sample size of this study. What more accurately depicts the validity of each approximation is the percent error compared to the manual approximations. The ellipse approximation most accurately predicted the spinal canal area. The accuracy of the ellipse approximation decreased as measurements moved caudally. An opposite phenomenon was observed for the triangle approximation. The change in relative error seems to suggest that the shape of the spinal canal changes from L1 to L5 as it moves caudally. This conclusion demonstrated by the approximations is consistent with current literature [10]. The lowest error of any of the approximations was the ellipse shape in L1. At this point, it had a percent error of 8.44%. This is comparable to the error observed in manual measurements of the spinal canal area due to partial volume effect, beam hardening, machine calibration, and spatial resolution at ±6% [16]. Another statistic to evaluate the accuracy of the approximation is to assess how correlated the approximation values were to the measured values. The correlation between each of the approximations and the measured values was incredibly high ranging from a value of 0.905 to 0.931. These values demonstrate the direct relationship between the IPD and the AP diameter approximations and the measured values.

When assessing the approximations as a whole, it is clear that they demonstrate a close relationship with the measured area of the spinal canal. Despite the close relationship, the variation in values may eliminate its use in clinical decision-making of lumbar spinal stenosis [17]. This is not only due to inaccuracy but also because the diagnosis of lumbar stenosis is made in the context of the whole clinical picture [18]. However, with the use of AI technology, these approximations could quickly be generated and assist in the quick assessment and judgment of patients presenting with lumbar spinal stenosis. In addition, additional mathematical analysis could be conducted to generate a second-order approximation which may predict the spinal canal area with greater accuracy still based on the IPD and AP diameter.

Limitations

This study is not without several limitations to consider. First, our findings may not be generalizable to patients outside of the exclusion criteria applied in this study. Furthermore, numerous instances of spinal stenosis present with a spinal canal deformity which adds a factor not accommodated by the approximation equations. Finally, the measurements were collected by several observers introducing additional error in approximations.

## Conclusions

The correlations between the approximation equations of the spinal canal described in this study and manually measured values of the canal are significantly related. The ellipse equation best predicted the area of the spinal canal followed by the triangle and then the rectangle approximation. The percent error difference of the ellipse approximation at L1 was similar in error compared to other causes of measurement error. Continued investigation into a second-order approximation may yield a more accurate approximation.
